# Perceptions and experiences of caretakers of children with neurologic deficits after severe malaria in Uganda

**DOI:** 10.1080/17482631.2026.2656560

**Published:** 2026-04-07

**Authors:** Allen Eva Okullo, Sheila Kisakye, Richard Idro, Michael Boele van Hensbroek, Chandy C. John, David Kaawa-Mafigiri

**Affiliations:** a Global Health Uganda, Kampala, Uganda; b Department of Social Work and Social Administration, School of Social Sciences, Makerere University, Kampala, Uganda; c Department of Paediatrics and Child Health, School of Medicine, College of Health Sciences, Makerere University, Kampala, Uganda; d Department of Paediatrics, Amsterdam University Medical Centers, University of Amsterdam, The Netherlands; e Ryan White Center for Pediatric Infectious Diseases and Global Health, Indiana University School of Medicine, USA

**Keywords:** Neurologic deficits, severe malaria, perceptions, experiences, child caretakers

## Abstract

**Purpose:**

Neurologic deficits may occur after severe malaria in childhood. This study sought to determine the perceptions and experiences of the caretakers of children with neurologic deficits after severe malaria in Uganda.

**Methods:**

This was a thematic analysis informed by a phenomenological interest in lived experience which involved twenty-three in-depth interviews and four focus group discussions. Interviews were audio-taped, transcribed verbatim, and back-translated into English for those conducted in the local languages. Data were coded and analyzed using content thematic analysis.

**Results:**

Neurologic deficits included motor, movement, cognitive, behavioral, and sensory deficits as well as epilepsy. Eight themes emerged which included: mixed perceptions about the deficits after severe malaria, difficulty in educating the impaired children, mistreatment by the community, mental and emotional distress, financial strain, misconceptions, misunderstandings and conflicts, limited access to specialized care services, and adaptation to a new way of life.

**Conclusion:**

Caretakers have misconceptions about the cause of neurologic deficits in their children. Caretakers have negative experiences, however, they learn to cope with their children’s impairments. Health messaging and education campaigns targeting caretakers of children with neurologic deficits within communities should be conducted to provide awareness of the neurologic deficits of severe malaria.

## Introduction

Neurologic deficits may occur in children with severe malaria (Boivin et al., 2007; Boubour et al., 2020; Trivedi & Chakravarty, 2022) with approximately 6–29% of children with cerebral malaria (CM) having neurologic deficits at hospital discharge (Trivedi & Chakravarty, 2022). Uganda, which ranks third highest globally in the burden of malaria (WHO, 2024), is disproportionately affected by malaria, with rural populations bearing the highest burden for several reasons, such as lower access to healthcare facilities, poverty, and cultural practices and beliefs that hinder access to care (Muganga, 2011; Omukisa, 2025). This, in turn, may predispose these populations to a higher burden of neurologic deficits after childhood with severe malaria.

These deficits may include conditions such as epilepsy; cerebral palsy; and cognitive, behavioural sensory, and motor deficits. Cognitive deficits reported after childhood severe malaria include learning impairments and poor memory (Boivin et al., 2007; Mbale et al., 2017), behavioural deficits such as attention deficits (Boivin et al., 2007; Opoka et al., 2009) and aggressive behaviour (Mbale et al., 2017). Motor deficits such as plegia and paresis (Idro et al., 2006; Oluwayemi et al., 2013), sensory deficits such as hearing and visual impairments, and epilepsy (Mbale et al., 2017) have also been reported. Unlike motor and sensory deficits, cognitive deficits are subtle, as has been reported in other studies (Bangirana et al., 2011; Holding & Snow, 2001) and become more obvious as children get older and start having academic challenges (Oluwayemi et al., 2013). Some of these deficits may resolve over time, but others may persist, ultimately affecting the quality of life of affected children as well as that of their caretakers, families, and communities.

Perceptions of illnesses, including neurologic deficits, are influenced by the sociocultural environment. Studies have revealed cross-cutting cultural differences in the perception and interpretation of factors associated with neurologic deficits such as epilepsy (Kaddumukasa et al., 2019). In many parts of Africa, superstitions exist regarding diseases including neurological disorders such as epilepsy (Ojinnaka et al., 2019). Epilepsy has been associated with either evil spirits or witchcraft (Kiwanuka & Anyango, 2018; Ojinnaka et al., 2019), fostering a preference for traditional and alternative healing systems for biomedicine among patients and their caretakers (Kiwanuka & Anyango, 2018). This renders it a non-biomedical problem rather than a medical problem that cannot be cured medically (Ojinnaka et al., 2019). Thus, in some cases, health-seeking behaviours for epilepsy and other neurological deficits have been found to be poor, even in the presence of readily available rehabilitation services.

Parents of children with neurological disorders experience socioeconomic stressors and exhaustion (Mungo et al., 2007). This may include conflicts in marriage due to the stress of blame, guilt, and anxiety; inadequate social support; and limited social life due to fear of rejection by family and friends (Vijesh & Sukumaran, 2007). Stressors also include challenges due to expenses related to the treatment and rehabilitation of children with disabilities. It is more expensive and time-consuming to raise a handicapped child with little prospects for the child to earn a living on his or her own (Lawal et al., 2014).

In a study conducted by Ansari in 2016, the presence of a child with neurodevelopmental disabilities affected all family members, most notably the mother’s life and health (Ansari et al., 2016). In this study, the mothers were overworked, had to perform all household work, and helped the disabled children with activities of daily living. In addition, most caretakers of these children face social stigma and discrimination, resulting in stress. Impaired quality of life in siblings of children with neurodevelopmental disabilities has also been reported (Ansari et al., 2016). In the case of epilepsy, caretakers tend to be in a state of uncertainty, apprehension, and need for constant surveillance, and often need to learn to cope with special diets, medication, challenges with schooling, and repeated hospitalisations (Rani & Thomas, 2019).

There are still unmet needs in the area of essential services and support for the lives of children with disabilities along with their caretakers (Ansari et al., 2016). These unmet needs are further exacerbated among low-income families, with parents consistently reporting an urgent unmet need for respite care, caregiving support, household assistance, and accessible child care (Ansari et al., 2016).

Given the burden of malaria in Uganda and neurologic deficits that have been reported to occur after severe malaria in childhood, this study sought to explore the perceptions and experiences of caretakers of children with neurologic deficits after severe malaria in Uganda. This will shed light on the beliefs and plight of these caretakers, which may inform social behavioural change communication interventions aimed at preventing, controlling, and rehabilitating neurologic deficits after severe malaria.

## Method

### Study design

We conducted a thematic analysis informed by a phenomenological interest in lived experience (Neubauer et al., 2019) of caretakers of children with neurologic deficits after severe malaria while controlling for any bias that may arise from the investigators’ own lived experiences (Creswell, 2013). We conducted 23 in-depth interviews (IDIs) and four focus group discussions (FGDs) with caretakers of children with neurologic deficits and seven IDIs with clinical staff involved in the care of children with neurologic deficits following severe malaria between September 2023 and March 2024.

### Participants and recruitment

Purposive sampling was used to select participants who met the eligibility criteria. Participants selected into the study included caretakers of children who had neurologic deficits 4 to 20 yrs after an episode of severe malaria, selected from a prior study (Malaria Impact on Neuro Development, MIND) conducted at the Global Health Uganda (GHU) office, and clinical staff managing neurologic deficits after childhood severe malaria from two hospitals serving a wide population including where the study participants come from. Clinical staff from both hospitals, who, besides offering care to children with neurologic deficits, interact with their caretakers, were in a good position to provide more information on the perceptions and experiences of these caretakers. Caretakers of children who had neurologic deficits 4 to 20 yrs after an episode of severe malaria from the MIND study but could not be reached via phone call, were excluded from the study. GHU is a non-profit organisation that conducts research in child health, neurodevelopment and interventions.

A neurologic deficit in this study was defined as an abnormal neurologic function of a body part due to injury of the brain, spinal cord, muscles or nerves that feed the affected part (Deluca & Griggs, 2020). Neurologic deficits among the children selected from the MIND study included: epilepsy, impaired hearing, movement disorders, and motor deficits, such as abnormal coordination, abnormal muscle strength of the upper extremities, abnormal muscle strength of the lower extremities, swallowing difficulty, abnormal facial symmetry, abnormal gait and station, ptosis, and paresis or plegia.

An invitation to participate in the study was sent via phone to caretakers of children with at least one neurologic deficit 4 to 20 years after severe malaria in the MIND study and to clinical staff at both hospitals. The MIND study from which the caretakers of children with neurologic deficits were obtained, found 72 participants to have neurologic deficits, out of a total of 1020 assessed four to 20 years after a childhood severe malaria episode. A contact list of the caretakers of these participants was created by reviewing the individual hard copy files of each participant with a neurologic deficit in the MIND study. A phone call was then made to each caretaker inviting them to participate in the study.

Upon acceptance, arrangements were made to transport each participant to the study site. All participants provided written informed consent before enrolment in the study. No participant was discriminated against from the study on the grounds of gender, disability, socioeconomic status, religion, or any other aspect. All participants consented to participate in this study.

**Figure 1.**
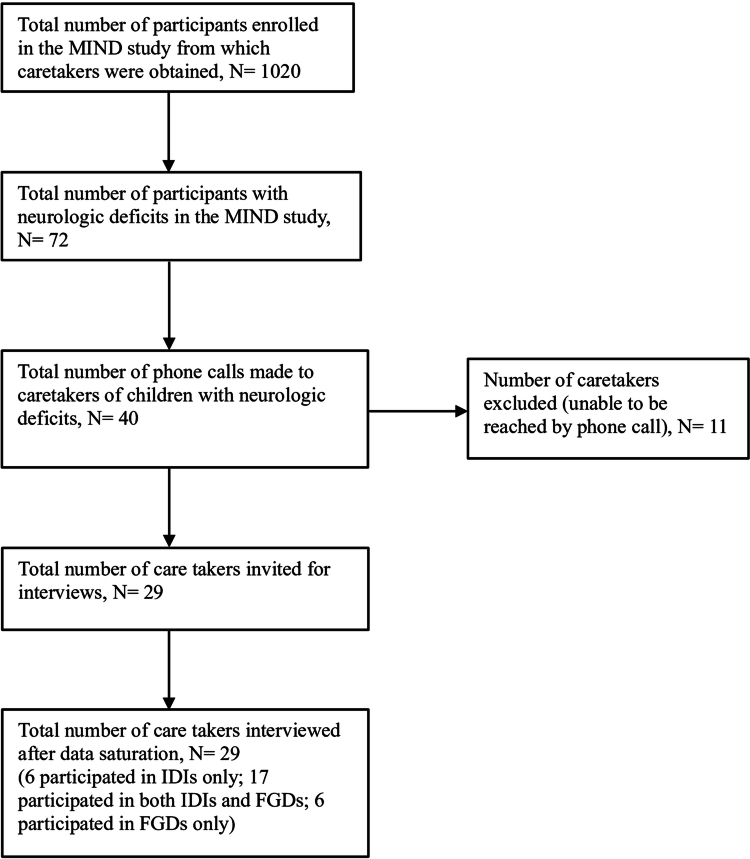
Selection of caretakers into the study.

A total of 29 invitations were made to the child caretakers out of the 40 phone calls made (caretakers could not be reached in 11 of the 40 phone calls made either due to permanently unavailable contacts or due to changes in phone contact) (Figure 1). No additional invitations were made following data saturation from the IDI’s and FGD’s conducted (Creswell, 2014).

### In-depth interviews (IDIs) and focus group discussions (FGDs)

IDIs were conducted at the GHU office, and at the two hospitals where the clinical staff participants were obtained, whereas FGDs were conducted at the GHU office. IDIs were chosen inorder to collect as much information as possible on the thoughts, perceptions and experiences of each caretaker in a focused setting with minimal distractions while FGDs were used to supplement the information derived from the IDIs by illiciting more responses by presenting an opportunity for interactions between caretakers (Creswell, 2013). The total number of 23 IDIs and four FGDs consisting of a total of 23 participants (17 of whom also participated in IDIs) (Table I) among the child care takers and seven IDIs among the clinical staff was determined following the principle of saturation as described for phenomenological studies by Creswell (Creswell, 2014).

**Table I.** Characteristics of the child caretakers and their children with neurologic deficits.

Caretaker data								Child data		
ID No.	Type of interview	Sex	Age (yrs)	Marital status	Education level	Economic activity	Relationship to child	Age (yrs)	Primary deficit in each child used to select caretakers for interviews	Other conditions identified by caretakers during interviews
1	IDI	M	66	Married	Secondary (S2)	Farmer	Grandfather	16	Cerebral palsy, cannot speak, abnormal facial symmetry, abnormal muscle strength upper extremities, abnormal coordination, abnormal gait and station, paresis/plegia	
2	IDI	M	58	Married	Post Graduate	Head Teacher	Father	15	Epilepsy/seizure disorder	Cannot see, talk, hear, walk
3	IDI	F	45	Married	Graduate	Not employed	Mother	15	Epilepsy/seizure disorder	Cannot see, talk, hear, walk
4	IDI & FGD	M	43	Married	Primary (P6)	Fishing & farming	Father	13	Abnormal coordination	Had hearing loss
5	IDI & FGD	F	31	Married	Primary	Farmer		7	Paresis/plegia	Learning difficulties
6	IDI & FGD	F	29	Married	Primary	Tailor	Mother	6	Abnormal coordination	Forgetfulness/poor memory
7	IDI	F	54	Single		Subsistence farming	Mother	13	Abnormal coordination	Slow in class, had epilepsy
8	IDI & FGD	F	36	Married	Primary	Farmer	Mother	6	Paresis/plegia	Epilepsy
9	IDI & FGD	M	30	Single	Illiterate	Business	Father	7	Abnormal coordination	Forgetfulness
10	IDI	M	37	Married	Secondary (S2)	Farmer	Father	8	Epilepsy/seizure disorder	Mental retardation
11	IDI & FGD	M	45	Married	Not educated	Farmer	Father	13	Epilepsy/seizure disorder, paresis/plegia	Paralysis on one side, cannot speak
12	IDI	F	49	Married	Secondary (S3)	Sells stones	Mother	17	Abnormal coordination	Child could not walk up straight, very forgetful
13	IDI & FGD	F	35	Divorced	Secondary (S3)	Farmer (animals)	Mother	9	Abnormal muscle strength upper extremities, abnormal gait and station, paresis/plegia	Previously couldn’t walk, weakness in hands
14	IDI & FGD	F	50	Widow	Primary (P7)	Farmer	Mother	8	Ptosis	Epilepsy, poor eyesight, hearing loss, poor memory
15	IDI & FGD	F	48	Married	Primary (P4)	Farmer	Mother	12	Swallow difficulty	Sometimes says things that don’t make sense
16	IDI & FGD	F	40	Widow	Primary	Farmer	Mother	7	Paresis/plegia	Forgetfulness
17	IDI & FGD	M	52	Married		Farmer	Father	8	Paresis/plegia	Had poor memory
18	IDI & FGD	F	31	Divorced	Secondary (S4)	Farmer	Mother	8	Abnormal coordination	Poor memory, doesn’t hear clearly
19	IDI & FGD	F	42	Married	Primary	Business (sells fish)	Mother	9	Epilepsy	Severe headaches
20	FGD	F	34	Married	Graduate	Business (salon)	Cousin	14	Abnormal facial symmetry	Withdrawn and slow in understanding
21	FGD	F	53	Divorced	Primary (P7)	Small business	Mother	18	Abnormal muscle strength upper extremities	Slow in understanding, got crippled (cannot walk up straight/she is bent)
22	FGD	F	62	Widow	Primary (P6)	Farmer (animal)	Aunty	6	Hearing impairment	Hearing loss, slow in understanding in class
23	FGD	M	51	Widower	Secondary (S4)	Not employed	Father	17	Abnormal facial symmetry	Short tempered/anger issues
24	IDI & FGD	M	63	Married	Primary (P7)	Farmer	Grandfather	9	Epilepsy	Slow in understanding
25	FGD	F	34	Single	Secondary (S3)	School Matron	Mother	7	Abnormal facial symmetry	Slow in understanding
26	IDI & FGD	F	28	Single	Primary (P5)	Hotel support staff	Mother	10	Anormal coordination	Anger issues, aggressive behaviour
27	IDI & FGD	F	49	Single	Primary (P6)	Farmer	Mother	17	Abnormal facial symmetry, abnormal muscle strength upper extremities, abnormal muscle strength lower extremities, abnormal gait and station	Cannot stand up straight
28	FGD	M	48	Married	Primary (P4)	Builder	Father	12	Swallow difficulty	Forgetfulness
29	IDI & FGD	F	51	Married	Secondary (S3)	Business	Mother	18	Abnormal muscle strength upper extremities	Right leg is lame, mentally retarded, squinted eyes

The IDIs and FGDs with child caretakers were conducted by four trained research assistants qualified in the social sciences (S.K, S.N, A.S and R.N), conversant in the local dialects of Luganda and Lusoga which are commonly spoken by the study participants. The IDIs with clinical staff were conducted by the principal investigator (A. E. O). Two of the research assistants (A. S and R. *N*) had prior interactions with the study participants in the MIND study (Malaria Impact on Neuro Development) conducted at Global Health Uganda (GHU) offices, in which they performed cognitive assessments on their children. Given their prior interaction, these research assistants (A. S and R. *N*) facilitated in the recruitment of the study participants. The remaining research team members had no prior interactions or relationships with the study participants. Personal biases were limited in each interview by interviewers abiding by the interview guide, actively listening and allowing participants to express themselves fully without interruptions or making any assumptions. The diverse backgrounds of the research assistants in the social and clinical sciences helped to better explore different perspectives from the study participants. All the study staff had no prior personal experience of a child with a neurologic deficit after severe malaria. At the start of each interview, the interviewer briefly introduced herself and the reasons for the research to the participant. Two separate open-ended IDI interview guides were used to guide IDIs with caretakers and clinical staff, respectively, while an FGD guide was used to aid the FGDs. Both the caretaker IDI and FGD guides were pilot tested with five participants and revised prior to the study. For each IDI or FGD, the interviewer was assisted by a second research assistant who took notes. Confidentiality was protected in the FGDs by withholding names of each participant. Instead, each participant was given an identity number by which they were referred. No persons other than the researchers and study participants were present during the interviews. Whereas the study had in place procedures to support participants who experienced distress, no one experienced such distress during the interviews. In the event that distress would arise, the study was prepared to offer referral to the participants’ routine care facilities for counselling and other related support. Each IDI took an average duration of approximately 30-40 minutes while each FGD lasted for approximately 90 minutes. All interviews and discussions were audio-recorded and supplemented with handwritten notes taken by a notetaker to capture non-verbal cues. In instances where more clarity needed to be sought regarding certain responses from particular participants, follow-up phone calls were made by a research assistant (S.N.).

### Data analysis

Data from the IDIs and FGDs were transcribed verbatim and translated into English for those conducted in the local languages (Luganda or Lusoga) by trained research assistants S.K and S.N. Semantic evidence was checked by having two independent translators, one fluent in Luganda and theother fluent in Lusoga languages, blind to the source text (that transcribed verbatim), back translate the transcripts in English to the original languages (Luganda or Lusoga) in which each interview was conducted. We then compared the back translated text with the source text which helped us to detect any discrepancies in meaning and nuances which were then discussed and resolved between the independent translators and the research assistants S.K and S.N to ensure consistency between the source text and the text in English. The IDIs with clinical staff were conducted in English and transcribed verbatim by research assistants S.K and S.N. All analysis was performed on the transcripts in English.

Data were then coded deductively by three independent coders (A. E. O., S. K., and M. *P*. H) with *apriori* codes derived from the interview guide and inductively by critically reviewing the data collected, and analysed in NVivo version 14.0, using content thematic analysis, which enabled us to identify and report themes. Three lists of *apriori* nodes developed by the three coders, each derived from an interview guide, were used to develop a framework in the form of three codebooks (one for the IDIs with caretakers, one for the FGDs with caretakers, and one for the IDIs for the clinical staff). Each codebook was used to code the respective transcripts. Each of the coders coded the transcripts line-by-line and new nodes that emerged during coding were added to the list through discussion between the coders. Discrepancy between the coders were resolved through discussions on conflicting ideas as well as the reasons behind them. This was done until a mutual decision was made between all three coders on which nodes to use. Coding consistency between the coders was assessed by using audit trails, that is, the detailed records of coding decisions and changes made to the codebook which also ensured consistency over time. The lists of nodes were regrouped into larger categories as themes that emerged from the data. The data were carefully reviewed to search for outliers as themes and patterns emerged.

### Ethical considerations

Ethical approval for this study was granted by the Makerere School of Medicine Research and Ethics Committee (registration number Mak-SOMREC-2023-594) and by the Uganda National Council for Science and Technology (registration number HS3065ES). All participants provided written informed consent prior to enrolment in the study.

## Results

Participants in the study included 29 caretakers (17 participated in both IDIs and FGDs, 6 participated in only IDIs and 6 participated in only FGDs) of children with neurologic deficits after severe malaria (Table I) and seven clinical staff from two hospitals involved in the management and care of children with neurologic deficits after severe malaria (Table II). The interviews consisted of 23 IDIs with child caretakers, four FGDs each with five to eight caretakers in attendance, and seven IDIs with clinical staff. The caretakers were aged 28-66yrs and most were female (66%). Most caretakers (97%) attained a primary level of schooling. Most participants worked in the informal sector (86%), of which 64 percent were farmers. Neurologic deficits among the children comprised of movement and motor deficits such as inability to walk, inability to move certain body parts, abnormal posture; sensory deficits such as impaired hearing, impaired speech and sight; and epilepsy characterised by falls, stiffening, jerking and seizures; cognitive deficits such as poor memory, reduced level of understanding, and learning difficulties; and behavioural deficits such as aggressive tendencies (Table I). Eight themes emerged on the perceptions and experiences of caretakers of children with neurologic deficits after severe malaria: mixed perceptions about the sequelae after severe malaria, difficulties in educating the impaired children, mistreatment by the community, mental and emotional distress, financial strain, misconceptions, misunderstandings and conflicts, limited access to specialised care services, and adaptation to a new way of life.

**Table II.** Characteristics of clinical staff.

ID No.	Sex	Vocation
1	F	Nurse
2	M	Medical Doctor- Paediatrician
3	M	Medical Doctor- Paediatrician
4	M	Nurse
5	F	Nurse
6	M	Medical Doctor
7	F	Medical Doctor

### Mixed perceptions about sequelae after severe malaria

When caretakers noticed abnormal changes in their children following severe malaria, they attributed these changes to various causes. These included being a result of severe malaria illness or the side effects of treatment given for severe malaria. Others attributed the abnormal changes to witchcraft and other sociocultural beliefs, including inheritance from the clan or deceased relatives.

*‘I thought that her problem was due to that complicated malaria that she had suffered from previously.’ (Caretake ID.5)*

*‘Okay whenever you get a sick person you get a lot of thoughts that you can think of but for me, I got to understand that first of all, the disease was caused by fever secondly, I understood that the doctor made some of the mistakes because they injected this child with medicine that was not appropriate. That’s what I thought.’ (Caretaker ID.10)*

*‘At that time people were telling us that it was ‘yabwe’, that the problem was from the family clan. Things like that, and they would say, take the child back to his family clan and they would say that the problem was from our clan. And he had yabwe, a disease that attacks children affected by fever due to severe malaria.’ (Caretaker ID.7)*

However, interactions with healthcare workers dispelled most incorrect perceptions and beliefs.

*Okay there is her grandmother, she died when her condition was not good and we thought that she was the one who brought that. So we thought that that was the problem. That’s what old people would say, until we went to the hospital in Kamuli and they told us. (Caretaker ID.17)*

*‘Still there was a time I was like, maybe it wasn’t malaria and I was like, why would the child get sick to that level. So when I took her to the hospital they told me that it was nothing but malaria.’ (Caretaker ID.8)*

### Difficulty in educating the impaired children

Impairments such as poor memory, decreased level of understanding, inability to comprehend what is being taught, and speech and hearing impairments made it difficult to cope with learning and education requirements, which resulted in poor performance and regression at school.

*‘He has a reduced ability to concentrate, poor memory, struggles with learning and finishing homework, very poor grades in school and always takes the last position in performance.’ (Caretaker ID.6)*

*‘My challenge has been that of seeing my child always having to come last in class position, secondly, the more the child comes last in position, the more they lose interest in learning, so, my biggest challenge has been to counsel and convince him never to quit studying despite the poor performance in class.’ (Caretaker ID.9)*

Cognitive and other impairments including frequent epileptic attacks eventually led to school drop outs.

*‘We tried taking her to school but teachers would return her home every time she got into the condition of epileptic attacks. So, we removed her from school.’ (Caretaker ID.10)*

*‘He was slow in school and after the Covid epidemic, I did not return him to school. I took him for a course. He was very slow at understanding things and that is why he even got tired’ (Caretaker ID.29)*

*‘What he studied before he got sick in baby and middle class is where he stopped. By the way he got sick when he had just joined middle class. He was very fine during that time I will not just praise him but that’s what he was’ (Caretaker ID.3)*

*‘The girl no longer goes to school because she does not speak and yet there are no schools for people who cannot speak. And in case they are there, they must be expensive yet I don’t have that money’ (Caretaker ID.11)*

### Mistreatment by the community

Both caretakers and their children faced foul treatment from the community members. They were stigmatised, made fun of, gossiped about, and bullied, including physical beatings by both teachers and fellow students at school.

*‘But there are times people say that that child doesn’t understand as though she is mad. These hurt me but I have nothing to do about it’ (Caretaker ID.12)*

*‘The truth is that my child gets challenged when he goes to school especially when they have not understood his condition. They disturb him a lot at school, there are teachers and students who beat him until I talked to them to be fair to him and I also talked to teachers about his condition.’ (Caretaker ID.13)*

*‘The conditions and experiences I have gone through are not good. When the child pulls herself, sometimes people gather and they laugh. So that hurts me as a parent.’ (Caretaker ID.10)*

However, in some cases, caretakers of impaired children received support from community members. This was in terms of advice on care, emotional support, and material supply.

*‘Some people in our community have sympathised with us and they advise me on how to feed the child. They have advised me on how I can treat the eyes of the child. They also advised me on how to treat him when he got stroke. They have cared and given me advise especially the adults on how to go about with this condition so that the child can be alive’ (Caretaker ID.13)*

*‘Our neigbours cared during the first years and they would call to ask you how he is and come to visit us but now they stopped. Only once in a while one or two can check on you but now they stopped it’s been many years. And you cannot blame them but they were supportive’ (Caretaker ID.3)*

### Mental and emotional distress

Caretakers experience mental and emotional distress in coping with their children’s impairments. One caretaker described this experience as traumatic, affecting the entire family. Others mentioned that it affects work because of worry and causes sadness.

*‘Trauma, there came a time when I could not sleep, and even now my sleep is very limited. This is because you hear him crying and you wonder what has happened. Even if you have rested you cannot totally say that you have rested. They look at him, that maybe now he has fallen sick, so psychologically, you are all tortured, as a family; all of you as a family.’ (Caretaker ID.3)*

*‘The effect of that could be seen because there were the older ones who had even reached a time of going to the university. But all of them grew thin, not because they didn’t have food but because they had no peace, because when he would fall sick, they would all fall sick.’ (Caretaker ID.2)*

*‘Okay, what I have experienced, okay she keeps you worried that time when we had to search for her we noticed that it would even make you not work well. And also your thoughts not being organised because of the child.’ (Caretaker ID.10)*

### Financial strain

Caretakers revealed several challenges that led to financial strain, including costs in seeking specialised care and treatment, economic productivity time lost in catering to the needs of the impaired children which involved in some cases assuming full-time care of the impaired children, loss of jobs due to absenteeism from work, and additional costs in catering to the educational needs of the children.

*‘In terms of finance like you know, when you have such a sick child, the money you would have used to buy something else, you have to use to take her for treatment and you can go when some tablets are not available because she takes three types of medicine and you could reach there when one type is not available and they tell you to buy it. So, the money you would have used to buy something else, you use it to buy tablets. Every money you make, you invest in the child.’ (Caretaker ID.8)*

*‘So, the time you should have spent at work, you spend attending to the child. So, all your finances reduce. That’s what makes our earnings reduce because whenever time passes that means that you are also affected financially.’ (Caretaker ID.10)*

*‘There was a time I got a job as a teacher but I lost it because there was a time he got stroke and I spent two weeks without working. I returned when they had replaced me with another person because they could not wait for me for that long.’ (Caretaker ID.13)*

### Misconceptions, misunderstandings and conflicts

Impairments in the children brought about misconceptions, misunderstandings, and conflicts between caretakers and their spouses and community members. Healthcare workers also shared accounts of caretakers who had gone through conflicts in their marriages and, in some cases, separation due to challenges brought about by the impairments.

*‘With my husband, things were not easy even up to now, we separated because there was a misunderstanding and that is what influenced me to leave home. I told my husband that incase he is taking my child to witch doctors I will not follow that. And in 2020, we separated because I noticed that things had failed and I was like this child was given to me by God and he has lived because of God. Incase God decides for him to die he will but not to take him to traditional healers.’ (Caretaker ID.13)*

*‘So, I’ve seen quite a number of mothers and they say they are single because they lost their husbands because of these children. A huge percentage of mothers say the husbands either left them or they don’t give them support because they say they don’t like such children, they don’t make such children, they can’t father such.’ (Clinical staff ID.3)*

*‘Because other people if you hear them, they say that one was sacrificed for wealth; you know people like a lot of wealth, and you tell them also to go and sacrifice their people, so that they also get. So, the situation is like that. And also, some people wonder why we don’t hide our child because most people put people with such conditions in the bedrooms. But for us here, we stay with our child everywhere.’ (Caretaker ID.2)*

### Limited access to specialised care services

Caretakers had limited access to specialised care for their children. This was due to stock-outs of required medication at health facilities, high costs of transportation for care seeking, difficulty in transporting children who could not walk, lack of nearby specialised facilities, and lack of knowledge on where specialised services could be accessed.

***‘**But sometimes you would use your transport and when you get there, they don’t have the medicine and now you have to go to pharmacies to buy.’ (Caretaker ID.8)*

*‘Even sometimes transporting them is not easy, like those who have grown up, if you don’t have a vehicle, it is very difficult to transport them.’ (Clinical staff ID.5)*

*‘Getting to see the right person currently is a matter of the person you are seeing knowing where you should find that kind of care. The kind of care I am talking about, finding a neurologist, or a physiotherapist, speech therapist, those therapists that help in neurological, psychologists, they are not wide spread.’ (Clinical staff ID.7)*

### Adaptation to a new way of life

Caretakers learned to cope with and adapt to life with sequelae. They taught their other children how to care for the impaired, which involved adopting new skills such as patience and understanding. This enabled them to return to social activities.

*‘And there are his younger siblings, they are now the ones who look after him, and the other issue is also about patience. We have taught them and they also know that they for example, don’t give him food hurriedly. He wants you to wait for him to chew and swallow and then you give him some more.’ (Caretaker ID.2)*

*‘It affects your income; it affects your relationships. There came a time when there was no function we could attend. Where would you get that happiness? And what would you go to do there? It is until we said no, there is life beyond this. There is something we are missing as people and other people who think we can be with them, so that’s it. But its only that we have been very positive especially with heath workers because we have never gone back to tell anybody else that we have gotten a problem.’ (Caretaker ID.2)*

## Discussion

Several studies have described the perceptions and experiences of caretakers of children with neurological disorders or sequelae (Ansari et al., 2016; Boubour et al., 2020; Lawal et al., 2014; Mbale et al., 2017; Mungo et al., 2007; Rani & Thomas, 2019; Vijesh & Sukumaran, 2007), a few of which are specific to children who had cerebral malaria (CM) before these sequelae (Boubour et al., 2020; Mbale et al., 2017). The current study unveils the mixed perceptions and negative experiences of the caretakers of children with neurologic deficits after severe malaria in Uganda. Caretakers had mixed perceptions of the cause of these impairments, which could have been influenced by their way of life, cultural beliefs and practices. These mixed perceptions could hinder good health-seeking behaviour, which would further escalate the burden of these sequelae within communities. A number of negative experiences such as limited access to care, misunderstandings, and conflicts are also likely to negatively impact the control of these conditions within the community by discouraging health-seeking. This study will thus help inform social behavioural change communication interventions designed to prevent, rehabilitate, and control neurologic deficits after childhood severe malaria, and in so doing, help to reduce the burden of neurologic deficits in malaria endemic countries.

Caretakers had mixed perceptions of the cause of neurologic deficits. Some attributed their children’s impairments to severe malaria illness having noticed changes in their children immediately after the illness. This is similar to a study that was conducted in Uganda in which community members attributed mental illness to malaria among other factors (Shah et al., 2017) and a study in Malawi in which many families attributed seizures to cerebral malaria (Mbale et al., 2017). A few caretakers attributed their children’s deficits to the treatment received for severe malaria probably due to negative beliefs and lack of trust in the medication administered. Other caretakers attributed their children’s deficits to witchcraft, as reported in other studies (Ojinnaka et al., 2019; Shah et al., 2017). Some caretakers associated the sequelae with cultural reasons such as inheritance from a relative who had passed on or inheritance from the clan. Culture has been shown to greatly influence beliefs regarding illness and disabilities in the African context, thereby influencing care seeking (Mkabile et al., 2021).

One of the several challenges experienced by caretakers was educating their children. Several caretakers reported poor performance among their children with some having repeated classes and, in worst-case scenarios, dropping out of school. This was a result of learning difficulties, characterised by a low level of understanding and poor memory. This is similar to what caregivers have experienced in studies where they described their children as being left behind in school (Mbale et al., 2017) and having experienced disability stigma in classrooms where they are hardly given any extra support by the teachers, finally dropping out of school as a result (Boubour et al., 2020). In some cases where children could not communicate (i.e., hear or speak), some parents were left with no option but to withdraw their children from school out of the belief that there were no affordable options for them due to lack of individualised care in ordinary schools (Boubour et al., 2020). This is consistent with previous studies in which parents viewed schools for special needs children as being too expensive to afford (Mkabile et al., 2021) and, as a result, withdrew their children from school. Sequelae, such as epilepsy with frequent epileptic attacks, also caused parents to withdraw their children from school. In other cases, caretakers incurred extra costs by spending more on teachers to cater to their children’s needs.

Caretakers were discriminated against and abused by community members. However, in a few cases, they were supported by the community. As has been reported in other African studies on children with disabilities, caretakers and their children were stigmatised probably as a result of cultural beliefs regarding children with disabilities (Boubour et al., 2020; Mkabile et al., 2021). Caretakers reported having been abused along with their children including bullying inform of physical beatings, being made fun of and being talked ill of behind their backs as has also been reported in studies on impaired children (Boubour et al., 2020; Mkabile et al., 2021). This greatly distressed the caretakers. However, in some cases community members provided support to caretakers in the form of showing concern, sympathy, and advise on how to treat and care for their children as has been reported in another study in Uganda (Namazzi et al., 2020). Community members have been reported to provide support to caregivers of children with disabilities in different communities (Namazzi et al., 2020) showing that communities have probably been sensitised to a degree about false beliefs and that empathy still exists amidst the prevailing stigma.

Caretakers suffered from mental and emotional distress. The caretakers of children who had severe impairments such as cerebral palsy and inability to walk, speak, or hear, were particularly more affected and described their experiences as traumatic, worrisome, disturbing and sad. They expressed that not only them but their entire family had been affected, including their other children, to the extent of developing physical symptoms. This is similar to other studies that have reported high levels of mental pressure, stress, and sadness resulting from the disability itself and all the challenges to the impaired child and the life of the caregivers and their families that result from it (Boubour et al., 2020; Mbale et al., 2017; Mkabile et al., 2021).

Caregiving duties due to their children’s disabilities depleted finances among caretakers, as reported in other studies (Namazzi et al., 2020; Resch et al., 2010). Caretakers incurred heavy costs during visits to health facilities in search of specialised care for their children. This was due to high transportation costs and the expensive purchase of medication, which is consistent with other studies (Namazzi et al., 2020; Resch et al., 2010). This caused a financial challenge for caretakers who travelled long distances from rural areas in search of specialised care many of whom could not afford the high costs. Caretakers also experienced financial strain as a result of economic productivity time lost due to the time taken off work to care for their children, as shown in other studies (Boubour et al., 2020; Namazzi et al., 2020) while other parents left their jobs to assume full-time care of their children with special needs, greatly lowering their family income. This is consistent with other studies that show that parents of children with special needs sometimes decide to assume full-time care of their children (Mkabile et al., 2021) further exhausting their finances. Other caretakers lost finances as a result of losing their jobs because of parental responsibilities, as reported in another study (Resch et al., 2010).

Misunderstandings, conflicts, and misconceptions were common for caretakers of children with different forms of neurologic deficits after childhood severe malaria. These arose between primary caretakers, who were usually mothers and fathers, and between caretakers and community members. Within families, disagreements on where to seek care, what care to seek, and blame accorded to the mothers by fathers in households that were already strained financially, strained relationships, which in some cases led to marital breakages. This is similar to studies that have reported marital strains and breakdowns between parents of children with disabilities (Mbale et al., 2017; Mkabile et al., 2021; Mukumbya et al., 2024; Resch et al., 2010). This might have been due to stress inflicted on parents by the sequelae, increasing demands in terms of time and money, differences in cultural beliefs and practices, failure to accept responsibility and the need to feel correct. There were misconceptions among community members regarding the cause of illness some of whom accorded blame to parents due to cultural and spiritual beliefs as has been reported in other studies (Mkabile et al., 2021; Namazzi et al., 2020). Conflicts with community members also arose due to mistreatment and discrimination faced by the caretakers within the community as has been reported in other studies (Mkabile et al., 2021). However, in some cases, conflicts were sparked by the aggressive tendencies of impaired children toward other community children.

There was limited access to specialised care for impaired children. This was due to lack of facilities and information on where to get care for neurologic conditions especially within rural communities as has been reported in other studies (Boubour et al., 2020; Mkabile et al., 2021; Namazzi et al., 2020; Resch et al., 2010). This led to high transportation costs and difficulties in transporting physically impaired children in search of care services, as has been reported in another study (Boubour et al., 2020). Although effective interventions for these impairments exist in Uganda, access to them, along with the required human resources for their management, remains limited to specialised facilities (Namazzi et al., 2020). This presents a major challenge in terms of rehabilitation of patients who are largely found in rural areas.

Caretakers adopted a new way of life as a way of coping with impairments. Caretakers came to accept the condition of their impaired children and appreciate the life that they still had. They learned not to isolate the impaired children as community members would expect but to involve them in activities with the rest of the children. Two caretakers returned to take part in social engagements that had been forgotten following the development of impairments in their children. Caretakers also taught their other children how to look after their impaired siblings as a way of life.

This study had some limitations. Participants for this study were obtained from a cohort of children who developed neurologic deficits more than five years prior to the study. This could have led to recall bias, which could have deprived the study of important experiences. We made efforts to minimise this by using probes to obtain information on the areas that needed more details. Participants might have been biased in reporting their true perceptions of neurological deficits for fear of being judged. However, we tried to minimise this by asking what their initial thoughts were and if their thoughts have changed since then to allow them to feel free to share their perceptions prior to any medical interventions and if this might have changed. Much of the study population was limited to participants from the central and eastern regions of Uganda, which might have deprived the study of interesting experiences and perceptions from other regions that might have had differing cultural beliefs and practices. However, this study also had some strengths. Health care workers were interviewed in addition to child caretakers, which provided additional information and valuable insight on caretakers’ negative experiences and misperceptions given their close interaction with these caretakers during care. Two methods of data collection, in-depth interviews and focus group discussions were used to collect information from child caretakers, which facilitated triangulation, allowing caretakers to interact with other caretakers, reflect on, and clarify their thoughts. Focus group discussions, which comprised caretakers with similar experiences, could have been a favourable environment, encouraging each one to share their thoughts and experiences more freely.

## Conclusion

Caretakers of children with neurologic deficits have mixed perceptions regarding the cause of impairments in their children after severe malaria. However, following sensitisation by medical personnel, impairments are attributed to severe malaria. This calls for continuous health education to sensitise communities on impairments and disabilities that might result from diseases such as malaria to avoid any misconceptions and promote good health-seeking behaviour. Caretakers’ experiences were largely negative and comprised challenges due to impairments. These included challenges in educating their children, relating to the community, mental and emotional distress, financial stress, discrimination and abuse, misconceptions, misunderstandings and conflicts within the family and with the community, and limited access to specialised care services. However, in light of these challenges, caretakers have learned to cope with and adapt to impairments. Information on neurologic deficits due to common illnesses such as malaria and where to seek care should be included in information education communication (IEC) and behavioural change communication (BCC) materials at health facilities and in all health messaging and education campaigns within the communities to create awareness that may help reduce discrimination, abuse, misconceptions and misunderstandings, foster appropriate health seeking and enhance prevention and control. Care for children with neurological deficits should be provided in medical outreaches within communities in order to bring these services closer to the people, especially those in rural areas.

## Data Availability

Data will be made available upon reasonable request from the corresponding author.

## Glossary

Neurologic deficit- an abnormal neurologic function of a body part due to injury of the brain, spinal cord, muscles or nerves that feed the affected part.

Yabwe- epilepsy characterized by falls, stiffening of the limbs, muscle jerks and convulsions.
